# Using cognitive behaviour therapy to explore resilience in the life‐stories of 16 UK centenarians

**DOI:** 10.1002/nop2.44

**Published:** 2016-02-23

**Authors:** Nimmi Hutnik, Pam Smith, Tina Koch

**Affiliations:** ^1^ Department of Mental Health & Learning DisabilitiesSchool of Health and Social CareLondon South Bank University103 Borough RoadLondonSE1 0AAUK; ^2^ Nursing StudiesSchool of Health in Social ScienceThe University of EdinburghDoorway 6Medical QuadTeviot PlaceEdinburghEH8 9AGUK; ^3^ School of Nursing and MidwiferyUniversity of SurreyGuildfordGU2 7TEUK

**Keywords:** CBT, cognitive, resilience

## Abstract

**Aim:**

In 2010, we interviewed 16 UK centenarians about their lives and later published a paper on the socio‐emotional aspects of positive ageing. We were struck by their ability to ‘move on’ from difficult situations which we recognized conceptually as ‘resilience’. In the effort to understand aspects of resilience as portrayed in their stories, we re‐examined their data.

**Methodology:**

In the original study, we used participatory action research (PAR) for its storytelling and group process components. Here, we re‐examine data from the centenarians’ life stories using a cognitive behavioural therapy (CBT) lens.

**Design:**

We focused on the notion of resilience in the centenarians’ stories guided by CBT insights to analyse and develop psychosocial interpretations.

**Results:**

Resilience comprised the ability to frame difficult life events in positive terms, accept what cannot be changed, manage worry and anxiety effectively, develop psychological flexibility in the face of change and continually seek opportunities for growth and development. We suggest that these resilient behaviours may have contributed to positive ageing.

## Introduction

The increase in the ageing population and in particular the oldest old has contributed to the persistence of stereotypes which characterize older people as physically and mentally incapable and intellectually frail. These stereotypes profoundly affect the way older people are perceived and consequently treated and devalued by society and care professionals alike. One of the ways to combat ageism and reverse negative stereotyping is by taking older people's stories into the public domain. This has been one of the driving forces for storytelling with centenarians which has motivated and guided our study. In the spirit of a participative world view and primary healthcare principles our collaborative research methodology, participatory action research (PAR) allowed us to engage and work alongside our research participants in a meaningful and reciprocal way. Making a contribution to positive ageing in the field of health promotion was one of our aims to enable older people's voices to be heard and available to inform future policy and practice, nationally and internationally.

### Background

A centenarian is a person who has achieved the age of 100 years or more which in developed nations is the fastest growing segment of the population. There are currently more than 14,450 centenarians in the UK (Office for National Statistics 2015) but little is known about their daily lives. A report on *Living Beyond 100* (Serra *et al*. 2011) provides an overview of the national literature, observes demographic trends and concludes that planning for housing, health and social care in preparation for the increase in future numbers of centenarians living in the UK needs to happen today. A recent UK study by Hazra *et al*. (2015) analysed electronic health records to report on the health differences between the sexes in a population of centenarians. The results showed that although more women than men lived to be 100 the men who became centenarians tended to be healthier.

Most developed countries have a significant cohort of centenarians. Their numbers are on the increase which is related to poverty reduction, fewer childhood deaths, advances in health care including the development of vaccines and medications, safe water supplies and better public health. Research over the last forty years has tended to be on centenarians with a focus on bio‐medical aspects of ageing and population based social research such as the Okinawa Centenarian Study (ONS) in Japan (begun in 1975: www.okicent.org, accessed 17 December 2015, Willcox *et al*. 2008, Freeman *et al*. 2010) and the New England Centenarian Study in the USA (begun in 1995, http://www.bumc.bu.edu/centenarian, accessed 17 December 2015 Perls *et al*. 1999). Centenarian studies in Sardinia and other European countries have been reported by Poulain *et al*. (2004) and Poon and Cheung (2012).

But there is still a lack of qualitative research with centenarians asking such important questions as: What contributes to positive ageing? What can we learn from centenarians to improve their care and guide health policy and practice in the future? The inspiration for the current study came from participatory action research with 24 Australian centenarians (Koch *et al*. 2005) who were asked about their secret of living to be a hundred as an indicator of successful ageing.

## The study

The research team consisted of three academics (Koch, Smith and Hutnik) and a research associate (Turner). The university's ethics committee approved this study. Increasing our understanding of positive ageing was the main aim and we believed we could learn from centenarians. Using popular media, we recruited volunteers from Ireland, Wales, Scotland and England. We provided centenarians with information about the study and sent them a consent form, which they signed and returned to us prior to interview. We travelled around the UK and interviewed centenarians in their own homes or residential settings. Initially we asked each person: ‘tell us your story’. We heard stories from five men and eleven women. Often their families and/or friends participated in the interviews and feedback. Storylines were collaboratively agreed on as part of the PAR process. The time frame was one year starting in December 2009–end 2010.

### Storytelling as a research method

Guided by Koch *et al*.'s (2005) earlier PAR study with Australian centenarians, we were prepared to use storytelling and group processes interchangeably (Koch & Kralik 2006). We commenced with one to one interviews in 2009 and completed them in 2010. Each person was asked to tell us in their own words, something about themselves and the social context that had shaped their lives. Centenarians selected whatever was foremost in their minds and/or any aspect of their lives that they wanted to share. A conversational approach to interviewing was adopted although several semi‐structured questions were included to encourage story telling such as: What is it like to have lived to 100 years and beyond? What, in your opinion is the secret of positive ageing? What matters to you today? In analysis we listened again to the interview recording, read and reread the verbatim transcription and produced a story draft reducing 8000 words to 3000 privileging the person's own voice. The interviewer wrote the first story draft, which the research team members then discussed, edited and generated a consensus story line. The resultant story was returned to the centenarian and/or significant other who then changed text, corrected, added and validated each story. In this way an evolving storyline was drawn together collaboratively as part of the PAR group process, where the group consisted of the centenarian, family, friends and the research team.

What did centenarians have in common other than their extreme age? Table 1 provides demographic details. Class, economic status, education and occupational differences were noted. Only four people were educated beyond the age of 15, all had married. Five were living in Nursing or Care Homes at the time of the interviews, while the others lived independently or with family. They were domiciled in Scotland, Northern Ireland, Wales and England.

**Table 1:** Sixteen Centenarians in 2010. Name, year of birth, age at leaving school, marriage year, living abode and birthplace

aName	Born	School	Married	Living	Birthplace
Olive	1908	14	1930	bNursing Home	London, England
Emily	1908	13	1932	Care Home	Rotherham Yorkshire
Hetty	1905	15	1932	cHostel	London, England
Albert	1908	14	1933	Own Home	Scarborough Yorkshire
Nita	1908	15	1933	Own Flat	Manchester, England
Alison	1907	18	1931	Own Home	Edinburgh, Scotland
Meg	1906	14	1938	Care Home	Dundee, Scotland
Minnie	1907	12	1936	Own Home	Ballywhisken, North Ireland
Jess	1908	15	1932	Care Home	Newcastleton, Scotland
Edward	1903	13	1928	Daughter	Cardiff, Wales
Bob	1908	21	1937	Hostel	Kingston on Hull, England
Phyllis	1908	18	1929	Own Home	Gibraltar
Alex	1909	15	1934	Own Home	Edinburgh, Scotland
Marion	1908	16	1936	Own Home	Edinburgh, Scotland
Jessie	1908	14	1940	Care Home	Aldershot, England
Frank	1910	12	1936	Hostel	Birmingham, England

a Centenarians wanted to be identified by their own name rather than by pseudonym.

b Nursing Home/Care Home where personal and nursing care is provided.

c Hostel provides independent living accommodation within an Aged Care Facility.

In the Australian centenarian study (Power *et al*. 2006, Koch *et al*. 2005, Koch & Kralik 2006; Koch *et al*. 2007) observed that centenarians seemed to embody positive attitudes and values but most importantly they had learnt to deal with stress in their lives. Similarly the UK centenarian cohort had experienced two world wars, societal upheaval, numerous life events, personal adversity and loss of close family and friends. We heard many stories of hardship, poverty and from our perspective, oppression. Longevity appeared not to be a result of avoiding stress rather it was responding to it efficiently and effectively. However, when talking about the stress and upheavals they had survived they said: ‘Accept whatever life brings’, don't worry about the past’, ‘take each day as it comes’, ‘do what you can to make things better and then forget it’, ‘give it time’ and ‘wait for things to change’. These findings were presented in previous publications (Koch *et al*. 2010a,b, Hutnik *et al*. 2012). In describing the centenarians’ ability to ‘move on’ from difficult situations, we recognized this response as ‘resilience’ which required further analysis. The purpose of the current paper therefore is to make an effort to understand and explore the centenarians’ stories in relation to ‘resilience’ by using a new lens, cognitive behavioural therapy (CBT), to re‐examine their stories.

Applying a Cognitive Behavioural Therapy (CBT) approach to re‐examine stories in the data in this paper we deliver an alternative interpretation of our data, by subjecting them to the specialist gaze of Nimmi Hutnik the psychologist and accredited cognitive behavioural (CB) therapist in our research team. Accordingly, to understand resilience as it is portrayed in the stories told by centenarians we re‐examined data using a CBT approach (Bannink 2012).

We reviewed current literature on resilience. Psychological resilience is conceptualized as the ability to confront adversity and still find hope and meaning in life (Padesky and Mooney 2012). Robertson (2012) Resilience is not just a ‘bouncing back’; it essentially incorporates ‘moving forward’ to make life better. This struggling from the pain of adversity towards ‘forward movement’ tends to place people who are resilient in touch with their own strongly held values and encourages positive movement in this direction despite difficulties and obstacles. Lavretsky (2014) in a comprehensive review of resilience and ageing research and practice suggests that spirituality, yoga, meditation, dance and movement therapies are effective interventions to increase resilience in the older population. Neenan and Dryden, advocates of CBT, point out that retraining the older person's attributional style in the face of adversity can facilitate the emergence of resilience (Neenan 2009:17).

CBT has become a unique psychotherapeutic approach to treating people with mental health issues that emphasizes the centrality of cognition in the determination of our feeling and behaviour. In everyday experience, the way we ‘frame’ a situation whether in positive or negative terms will determine the way we feel about it and how we behave in it. In CBT we encourage people to challenge our negative thoughts to develop more flexible, realistically optimistic ways of thinking about difficult situations. Recently new developments in CBT arose from the realization that merely challenging our negative thoughts is sometimes insufficient to bring people out of depression and/or anxiety, particularly in situations that are irreversible. Acceptance and Commitment Therapy (ACT) expands CBT, focusing on being mindful and aware of our thoughts and our relationship with them to enable ourselves to move in the direction of our most deeply cherished values despite irreversible situations or handicaps (Hayes *et al*. 2006). ACT is based on the Serenity Prayer: Give me the grace to accept the things I cannot change, to change the things I can and the wisdom to know the difference. In other words, intellect is required to think through and withstand life's difficulties. To understand how the centenarians in our study dealt with situations that potentially could have ‘knocked them down’ we used CBT as a theoretical lens to explore their stories to find and interpret positive ways of being in the world (Bannink 2012).

### Re‐examining storylines in the data to illuminate ways of being in the world

In practice CB therapists understand that while cultural and contextual problems can seem overwhelming and unsolvable to a person, alongside these unavoidable events there are several protective factors to be aware of when dealing with clients (Weerasekera 1996). Although Weerasekera talks about the ‘4Ps’ model of case formulation: predisposing, precipitating, perpetuating and protective factors, for this study, we are interested in protective factors which are summarized as: encouraging good relationships with family, friends and in a social circle; avoiding seeing problems as unbearable; accepting circumstances that cannot be changed; moving towards realistic goals; taking a decisive action in an adverse situation; after a loss looking ways to better understand the self; viewing problems in a broader context; maintaining hope; paying attention to one's physical and emotional well‐being. Once the stories had been returned to centenarians Hutnik, the CB therapist in our team focused on the cognitive and emotional content of their storylines to develop a psychosocial interpretation or commentary for each.

## Results

We learnt that centenarians had already learnt to modify their cognitions, emotions and behaviour when dealing with problematic or troublesome life vents. Centenarians had ordinarily and unwittingly: (1) mastered the art of positive framing; (2) learnt to accept things they could not change; (3) learnt to manage their worry and anxiety; (4) had developed psychological flexibility with regard to change and (5) had embraced opportunities to flourish. In the section below, we present the findings of re‐examining storied data.

### Mastering the art of positive framing

Re‐reading their stories we noted that the centenarians in our study regularly ‘framed’ difficult situations and events in positive terms. On numerous occasions, Olive put a positive spin on life: she counted herself as lucky to have escaped a large bomb that fell nearby, lucky that her husband, Herb, was not asked to leave the country during the war, lucky to have had a security button around her neck when she had a fall at home, where she had been living independently. She claimed to be lucky that husband Herb did not lose his speech when he had his stroke. Olive did not catastrophize her life events (Peterson *et al*. 1998). Indeed she did the opposite, looking on the bright side of life. When we visited Olive in a Nursing Home, we viewed her current situation as oppressive, being surrounded by many silent and older people suffering from dementia, yet Olive merely said:

Yes my life has changed but it has changed for the better, definitely upward. It is good in this nursing home because there's always someone around. The carers are very kind. It's quite a family atmosphere.

Other centenarians too demonstrated this ability to positively frame or reframe difficult life events such that the sting was taken out of them. Although centenarians had lived through difficult situations and hard times, most revealed that they did not view their lives as being particularly stressful. There seemed to be a matter of fact attitude to difficulties, losses and sadness that they considered to be part of ordinary life. They also described ‘accepting things they could not change’.

### Accepting things they could not change

Bob was a good example of this approach who talked about his ability to accept things he could not change:

I have a capacity for withdrawing into myself and letting the world go by… I had, as a child, a capacity to withdraw with a book, to a corner in a very busy house where everything was going on. And I could be lost to that world in a book, sitting in a corner behind the sofa, reading and everybody would say, ‘Where's Bob? Where's Bob? Come on, what are you doing there?’ But I had a capacity for retreating, if you like, psychologically, to a place where I was alone and I didn't have to worry about what was going on outside. So yes, I suppose it helped me to cope with what could have been stress, but I could easily say ‘well, there is nothing about this situation that I can do. I can't alter the fact that Japan has invaded or attacked Pearl Harbour. So I don't need to worry about it because it's not my business.

In another interview with Frank, who had turned 100 years of age in 2010, we observed that in his current housing situation he was responsible for the daily, physical care of his dementing 96‐year‐old wife, Mary. This responsibility deprived him of time to spend painting in watercolour, which is something he would have liked to do. We noticed a tinge of sadness and regret here but his narrative indicated that ‘he just got on with it’. The dogged ability to just ‘plod on’ in the face of life's many stressors was not an unusual attribute in the accounts we gathered.

Phyllis was matter of fact when she said:

… quite a lot of things have happened… different things, what with my father being killed and then my husband going to the war and my brother being killed and things like that. But then again, you're left and you've got to get on and that's it…You just have to cope with it, don't you? I'm afraid I'm one of those resilient people, I don't just sit down and cry when it comes, I've just got to get on with it.

Albert's motto was: ‘If you can't change it and alter it, don't worry about it’. Albert had worked in the coal mines since he was 14 and had severe lung incapacity as a result – yet at 102 this did not deter him from dancing regularly.

### Managing their worry and anxiety

We noticed that centenarians seemed to have developed effective strategies to cope with worry and anxiety, thus enabling them to bounce back when under stress.

In talking about how Minnie dealt with stress in her life she said ‘if you get worried, really worried about something, there's usually someone that you can talk things over with’. This was particularly significant for Minnie who had lived through the ‘Troubles’ in Northern Ireland.

Indeed Nita gave this advice:

If you can manage and you've not got pain, you've got to try and push worry away and not be miserable. Try to be happy and try and join in every day with what's going on. Accept what comes. Think about being thankful to be alive.

### Developing psychological flexibility with regard to change

Many older people seek to maintain the familiar and are resistant to change. However, the participants in our study seemed to have developed a flexible attitude to change. Olive for example made some significant changes in her life: she adjusted to the loss of her property, moving from a very large house into a very small and noisy one with her two young children; in later life, she moved into her daughter's house after her fall and then into the Nursing Home, a transition from independence to dependence that many older people find hard to make. At the time of interview, she was adjusting to the changes that physical frailty brings. Although she told us that had she one wish, it would be to regain the use of her legs, her attitude was one of equanimity rather than complaint.

Indeed some participants actively sought change to enable them to deal with adversity and loss. Jess attributed her resilience to determination and to making changes in her life. She said: ‘Just determination sometimes made me move on and a very good thing to do is to change things’. Jess's daughter who was present at her interview gave an example of how after her mother was widowed, ‘you immediately started to do things in the house and move things and change things, created new bits in the garden and all sorts. So you actually applied yourself when you were under stress, to tackle new things’. Jess agreed ‘oh yes, it's a very good thing to do, is to change things’. Thus, psychological flexibility in the face of change seems an ingredient of resilience.

However, Alison was resistant to change, particularly the demands that new technology made on her with computers, emails and answerphones. She was grateful that she had a daughter who could deal with the latest communication technology and in this way she could still keep in touch with others in her small social group.

### Embracing opportunities to flourish

Centenarians in our study regularly sought opportunities to flourish (Seligman 2011). Olive, even in her nursing home, kept an active interest in the world through the television. For Hetty the achievement of World Peace was a primary value: at 100 she had marched against the Iraq war in the London streets. Emily campaigned against the closure of her nursing home and was featured on BBC news. Alex at a 100 was the Honorary President of the local Bowling club and an active member of the Free Masons. Phyllis’ passion for bridge led her to start and develop the local Bridge Club, which had grown to three hundred members strong. Edward, already a centenarian in 2005, was awarded the Welsh Good Neighbour Award and the Neighbour from Heaven Award for turning his garden into a playground for children and adolescents. Albert learnt Tai Chi at 101. Bob's childhood passion for reading books had continued into his 100s. Marion was busy planning another ceilidh 3 years ahead for her 105th birthday. Meg, who is now 103, had continued house cleaning as a paid job until she was 82. Nita swam every day at the local pool until she was 101. Jess wanted to keep on travelling although she was 101. Alison, 102, had recently been awarded an OBE for her exquisite glass engraving skills. Each of these centenarians had a passion that brought them fun and joy and kept them in contact with other people or with contemporary issues and ideas. They were active in their contribution to others and held fast to the belief that life is worth living and that their lives had meaning (Koch *et al*. 2010a,b, Hutnik *et al*. 2012).

Marion said:

Never give up. I don't think of death. I think of living and what I am going to do and what I am going to enjoy.

We found it significant that very few of those who participated in the study mentioned death. It was as if they wanted to ‘drink life to the lees’, a quote from Tennyson (1842) in the sense that they wanted to live life to the full signified by drinking a glass of beer or wine to the very last drop so that only the dregs (lees) remained.

## Discussion

At the heart of CBT is the tenet that the way we think about things determines the way we feel about them (Beck *et al*. 1979). Troy *et al*. (2010) suggest that reappraising the meaning of a difficult situation so that it is seen in more positive terms results in an adaptive and resilient response. John and Gross (2004) show that people who regularly use positive framing or cognitive reappraisal as a mechanism to cope with stress report greater psychological well‐being than those who do not. Many people automatically think negative thoughts about a situation causing them to feel down and anxious and to behave in ways that are counterproductive to wellbeing. Greenberger and Padesky (1995) have developed a powerful tool, the thought record, to help people challenge those negative automatic thoughts so as to arrive at an alternative or more balanced way of thinking about the situation. Seligman (2002) encourages the conscious choice to develop a more positive explanatory style as a means towards lasting fulfilment. It is enlightening to realize that centenarians had developed long‐term habits of positive framing difficult situations. This ability stood them in good stead whenever life was stressful.

The scientific literature suggests that acceptance is a key ingredient in tolerating highly stressful situations among extreme environmental hardship and threats to life (Siebert 1996). Silver *et al*. (2002) found reduced levels of post‐traumatic stress disorder among individuals who ‘accepted’ the terrorist attacks of September 11 2001. Among mothers whose children had been diagnosed with a life threatening cancer, Manne *et al*. (2002) found less depression among those who accepted the situation. Acceptance is a cornerstone of ACT (Hayes *et al*. 2006). Centenarians demonstrated resilience in accepting the things that they could not change without anger or self‐pity and with a determination to cope with whatever life threw at them.

Many centenarians, for example, Hetty and Nita, talked about being able to sleep very well at night because they could leave their concerns behind. This indicated to us that part of resilience is the skill of managing worry and anxiety. Espie *et al*. (2012) suggest a ‘wrapping up’ exercise at the end of the day to deal with sleeplessness. This involves journaling about what has been difficult and what has been good and developing a ‘to do’ list for the morrow. The exercise ends with a closing of the journal and a determined refusal to ruminate on stressors and problems. It would seem that both Hetty and Nita had unwittingly learnt this skill.

A potent tool used by CB therapists is the Worry Tree (Butler & Hope 2007) where anxious people are asked to notice what they are worrying about and then to ask themselves if they can do something about it. If the answer is yes, then they are instructed to make an action plan about what to do, when to do it and how to do it. Then they are encouraged to let the worry go and change their focus of attention. If they cannot do anything about it then they are encouraged to let the worry go and change their focus of attention. We did not ask centenarians if they had received counselling in their long lifetime. Apparently intuitively, some of the centenarians in our study had picked up this skill in dealing with stress. We suggest that the effective management of worry and anxiety lays the foundation for the ability to be resilient and to bounce back in the face of stress.

According to ACT (Hayes *et al*. 2006) the root of much of our psychological pain is inflexibility. When we learn to develop psychological flexibility we increase the level of our happiness and well‐being. Centenarians appeared to be temporarily shaken by their losses and then they adapted. They were able to let go of the way things used to be and they reoriented themselves to the way things are now. Thus, having the psychological flexibility (Hayes *et al*. 2006) to adjust positively to change seems key to being resilient. Indeed, some actively sought and created change as a means of dealing with adversity.

Resilience usually means speedy recovery from problems, the ability to recover quickly from setbacks (Netuveli *et al*. 2008). We did not specifically ask centenarians how they recovered and moved on – we interpreted that they do so from what they said ‘just get on with it’ or ‘plod on’ regardless and so on. Of course this could be a British and/or generational characteristic. We believe that the context where they grew up and aged is important. It is with caution that we say that resilient people have an ability to identify and respond effectively to a potentially disruptive situation (Atkinson *et al*. 2009). The debate in the resilience literature about whether the term resilience merely implies ‘recovery to stability’ or whether it may include the concept of growth and evolution following a disruptive event continues. We agree with Seligman (2011), the father of positive psychology, who suggests that ‘flourishing’ is an essential ingredient of resilience. These centenarians reported that their reason to rise in the morning was to spend some of that day doing something interesting to further their growth and development.

### Study limitations

The main limitation of our study was its small‐scale qualitative nature and self‐selected volunteers who were not representative of the centenarian population at large. The study design addressed the limitations of small‐scale qualitative research by employing participatory action research which involved the centenarians and their families in co‐constructing their stories to generate rich in‐depth data to which a range of theoretical and analytical lenses were applied.

## Summary and conclusion

We have shown that 16 centenarians demonstrated resilience in their ability to positively frame very difficult life events by considering themselves ‘lucky’ or ‘fortunate’ thereby creating for themselves positive emotion. Their resilience was also evident in their ability to accept things they could not change (such as physical decline) with resignation and at the same time, a determination to move on. They knew how to manage anxiety and worry. Additionally, resilience was seen in their psychological flexibility, their positive attitude to and adaptation to change. We also found resilience in their quest to flourish, to be engaged in their passions, hobbies and interests and thus to find continuing meaning in their very long lives.

What is the impact of these findings on health policy and practice? We believe we have uncovered some key ingredients of resilience in the very old. What can we do with this information? We suggest that everyone already has some resilience and we can all learn to improve our resilience capabilities. In a limited sense, healthcare professionals may be able to help older people accept the things that they cannot change (such as physical deficits e.g. restricted mobility, failing eye sight). By realizing social connection and activity are important for health we may encourage our clients/patients to engage in these interests as a way of enhancing resilience.

Work in this field continues as the first author is in the process of developing a Resilience Awareness Training Tool (Hutnik, In Press) that can be used by healthcare professionals to develop and build resilience in their clients/patients. The next phase is to provide CBT guidelines for positive framing of difficult life events and how to help clients/patients manage anxiety or develop psychological flexibility in the face of change. Further research is required to explore generational cohort factors such as the value of ‘just getting on with it’ and ‘keeping a stiff upper lip’.

Although research shows the prolonged transition from active independent living into chronic ill health, frailty and eventual death in older people may be experienced among care staff as ‘living bereavement’ (Holman 2008), a more positive picture emerged from our centenarian study which contradicted these negative stereotypes. Our analysis demonstrates that emotional resilience is clearly a resource present in older people which can be tapped by nurses and other care professionals to jointly plan care to promote independence and personhood.

What began as a quest to understand what is extraordinary in the lives of centenarians has revealed the power of the ordinary. It seems to us that resilience is indeed an ‘ordinary magic’, a term coined by Masten (2001). We understand resilience to be a complex interplay between biology, psychology, culture and environment and a powerful resource with the potential to promote the health and wellbeing of older people.

### Application to clinical nursing and other care professionals

Recognizing the centenarians’ resilience in overcoming adversity and its contribution to positive ageing can assist nurses and other care professionals to overcome the negative stereotypes frequently associated with caring for older people. Furthermore it can enable professional carers to tap older people's resilience as a resource to jointly plan care and promote independence and personhood.

## Author contributions

All authors have agreed on the final version and meet at least one of the following criteria [recommended by the ICMJE (http://www.icmje.org/recommendations/)]:

- substantial contributions to conception and design, acquisition of data, or analysis and interpretation of data;
- drafting the article or revising it critically for important intellectual content.
